# Oncogenic Mutant *p53* Sensitizes Non–Small Cell Lung Cancer Cells to Proteasome Inhibition via Oxidative Stress–Dependent Induction of Mitochondrial Apoptosis

**DOI:** 10.1158/2767-9764.CRC-23-0637

**Published:** 2024-10-15

**Authors:** Kranthi Kumar Chougoni, Victoria Neely, Boxiao Ding, Eziafa Oduah, Vianna T. Lam, Bin Hu, Jennifer E. Koblinski, Bradford E. Windle, Swati Palit Deb, Sumitra Deb, Jorge J. Nieva, Senthil K. Radhakrishnan, Hisashi Harada, Steven R. Grossman

**Affiliations:** 1 Department of Medicine, Keck School of Medicine of USC, USC Norris Comprehensive Cancer Center, University of Southern California, Los Angeles, California.; 2 Philips Institute for Oral Health Research, VCU School of Dentistry, Virginia Commonwealth University, Richmond, Virginia.; 3 Division of Medical Oncology, Department of Medicine, Duke University School of Medicine, Duke Cancer Institute, Duke University, Durham, North Carolina.; 4 VCU Cancer Mouse Models Core, Virginia Commonwealth University, Richmond, Virginia.; 5 VCU Massey Comprehensive Cancer Center, Virginia Commonwealth University, Richmond, Virginia.; 6 Department of Pathology, VCU School of Medicine, Virginia Commonwealth University, Richmond, Virginia.; 7 Department of Biochemistry and Molecular Biology, VCU School of Medicine, VCU School of Medicine, Virginia Commonwealth University, Richmond, Virginia.

## Abstract

Non–small cell lung cancer (NSCLC) cells with oncogenic mutant *p53* (*Onc-p53*) alleles exhibit significantly higher levels of proteasome activity, indicating that Onc-p53 induces proteotoxic stress which may be leveraged as a therapeutic vulnerability. Proteasome inhibitors (PI) are most active in cells under proteotoxic stress, so we investigated whether PIs exhibit preferential cytotoxicity in *Onc-p53* NSCLC cells. Indeed, bortezomib (BTZ) and other PIs exhibited IC_50_ values 6- to 15-fold lower in *Onc-p53* cells versus *wild-type (WT) p53* cells. BTZ cytotoxic effects in *Onc-p53* cells were abrogated by antioxidants such as *N*-acetyl-L-cysteine, indicating that oxidative stress is the critical driver of BTZ-dependent cytotoxic effects in *Onc-p53* cells. Importantly, we observed oxidative stress–dependent transcriptional induction of the proapoptotic BH3-only protein NOXA, leading to cleavage of caspase-3, consistent with an apoptotic mechanism of cell death in *Onc-p53* but not in *WT p53* cells treated with BTZ. BTZ-generated oxidative stress was linked to nuclear translocation of NRF2 and transcriptional activation of ATF3, which in turn was required for NOXA induction. Validating the translational potential of BTZ in *Onc-p53* NSCLC cells, BTZ and the BH3-mimetic navitoclax were synergistically cytotoxic in *Onc-p53* but not in *WTp53* cells *in vitro*, and BTZ effectively limited the growth of *Onc-p53* NSCLC xenografts when combined with navitoclax and carboplatin (a standard-of-care chemotherapeutic in NSCLC) *in vivo*. Our data therefore support further investigation of the therapeutic utility of PIs combined with BH3-mimetics and chemotherapy in *Onc-p53* human NSCLC as a novel therapeutic strategy.

**Significance:**

NSCLC is the leading cause of cancer death due, in part, to a lack of active therapies in advanced disease. We demonstrate that combination therapy with a proteasome inhibitor, BH3-mimetic, and chemotherapy is an active precision therapy in NSCLC cells and tumors expressing *Onc-p53* alleles.

## Introduction

Lung cancer is the most commonly diagnosed cancer affecting both men and women, and non–small cell lung cancer (NSCLC) is the most common histologic subtype of human lung cancer (84%), which is the leading cause of cancer-related death annually in the United States (1, 2). There has been substantial progress in deciphering the molecular underpinnings of lung oncogenesis and tumor progression, including the complex contribution of alterations in the p53 pathway (3). Whereas loss of p53 function through alterations in the *p53* gene or in genes encoding components of the p53 pathway (*MDM2*, *p14ARF*, etc.) is nearly universal in human cancer (4), nearly 70% of NSCLC tumors retain a mutated *p53* allele encoding a missense mutated or truncated p53 protein that may display emergent oncogenic functions (hereafter noted as “*Onc-p53*”; 5–9). Thus, the frequent finding of NSCLC tumors retaining *Onc-p53* alleles offers the potential to exploit *Onc-p53*–associated cancer cell–specific vulnerabilities for therapeutic benefit (5, 10).

One such potential vulnerability is proteotoxic stress, evidenced by heightened proteasome activity, observed in many tumor cell types expressing Onc-p53 proteins, including lung cancer (11, 12). Proteasome inhibitors (PI) induce cellular stress at multiple levels, including proteotoxic, autophagic, and oxidative stress, but are particularly effective in neoplastic cells with ongoing proteotoxic stress (13). However, the clinical utility of the PI drug class has been limited by lack of efficacy outside of B-cell malignancies, such as multiple myeloma (MM) and mantle cell lymphoma (MCL), which are uniquely sensitive to PIs because of immunoglobulin synthesis–related proteotoxic stress (14).

The mechanism of PI resistance of most solid tumors, including NSCLC, remains unclear, as there has been limited exploration of precision molecular techniques that might identify subsets of tumors sensitive to PI alone or combined with other targeted agents, as has been successfully deployed in MM. Relevant to known mechanisms of PI cytotoxic action, an additional vulnerability associated with *Onc-p53* expression in many tumor types is the presence of excess reactive oxygen species (ROS; 15). Excess ROS in the baseline state for cancer cells could theoretically sensitize them to therapeutics such as PIs that generate additional ROS to a level toxic to cancer but not normal cells due to additional deficiencies in ROS scavenging mechanisms (16, 17).

Based on previously described overexpression of proteasome subunits and excess proteasome activity in NSCLC cells expressing Onc-p53 protein (11), we investigated whether PIs exhibit preferential cytotoxicity in *Onc-p53* NSCLC cells *in vitro* and *in vivo*. Indeed, bortezomib (BTZ) and other PIs were preferentially cytotoxic in *Onc-p53*–expressing NSCLC cells which also exhibited higher levels of proteasome activity, indicating that Onc-p53 proteins induce proteotoxic stress. Importantly, we observed an oxidative stress–dependent transcriptional cascade in cells treated with BTZ, resulting in induction of the proapoptotic BH3-only protein NOXA and apoptotic cell death. Validating the translational potential of BTZ in *Onc-p53* NSCLC, combinations of BTZ with carboplatin or the BH3-mimetic navitoclax were synergistically cytotoxic *in vitro* and effectively limited the growth of mutant *p53*–expressing NSCLC xenografts *in vivo*. Our data therefore support further investigation of the therapeutic utility of PIs combined with BH3-mimetics and chemotherapy in human NSCLC tumors expressing *Onc-p53*, as novel therapeutic strategies are needed for the many patients with NSCLC who do not benefit from current targeted or immunotherapies.

## Materials and Methods

### Cell lines and drugs

A549 (*WT p53*; RRID: CVCL_1472) and H1703 (*p53*^G262fs^; RRID: CVCL_1490) cells were purchased from ATCC which verifies all cell lines by short tandem repeat polymorphism analysis. H1975 (*p53*^R273H^; RRID: CVCL_1511) and H1437 (*p53*^R267P^; RRID: CVCL_1472) cell lines were the generous gift of S. Deb (VCU), and H460 (WT; RRID: CVCL_0459) and H460-p53KO cells (18) were the generous gift of D. Gewirtz (VCU). H1975, H1437, H460, and H460-p53KO cells were verified by short tandem repeat analysis (Univ. Arizona Genetics Core Lab and Labcorp Cell Line Authentication Division). Cells were passaged a maximum of 2 months and were monitored for *Mycoplasma* contamination using the MycoStrip Mycoplasma Detection Kit (InvivoGen). All cell lines were maintained in RPMI 1640 media (Thermo Fisher Scientific, 11875093), supplemented with 10% heat-inactivated FBS (Corning, 35-010-CV) and 100 µg/mL penicillin G/streptomycin at 37°C in a humidified, 5% CO_2_ incubator. Bortezomib (BTZ; MedChemExpress, HY-10227 and LC Laboratories, B-1408), carfilzomib (CFZ; Selleck Chemicals, S2853), navitoclax (MedChemExpress, HY-10087), QVD-O-Ph (MedChemExpress, HY-12305), *N*-acetyl-L-cysteine (NAC; MedChemExpress, HY-134495), and glutathione ethyl ester (GSH-EE; MedChemExpress, HY-134124) were dissolved in DMSO; carboplatin (MedChemExpress, HY-17393, Selleck Chemicals, S1215) was dissolved in H_2_O, and stable drugs were stored at −20°C in the dark. The final concentration of DMSO was 0.1%.

### Generation of cell lines expressing short hairpin RNA, cDNA and CRISPR/Cas9 knockouts

Lentiviral short hairpin RNA (shRNA) vectors, shNOXA (TCRN0000338867), shNOXA#5 (TCRN0000338864), shNRF2 (TCRN0000273494), shATF3 (TCRN0000329690), and shATF3#3 (TCRN000013568), were purchased from Sigma-Aldrich. Scrambled shRNA (RRID: Addgene_1864), pLenti6-GFP (RRID: Addgene_35637), pLenti6/V5-p53_R273H (RRID: Addgene_22934), psPAX2 (RRID: Addgene_12260), and pMD2.G (RRID: Addgene_12259) were purchased from Addgene. Each plasmid was cotransfected into HEK293T cells (ATCC; RRID: CVCL_0063) with psPAX2 and pMD2.G using EndoFectin (GeneCopoeia, EF001). Lentivirus-containing supernatants were collected and were used to infect the cell line of interest, and stable cell lines were established by either 2 µg/mL puromycin or 1.0 mg/mL G418 sulfate selection. Control (#2) and p53 guide RNA (#4) vectors targeting human *TP53* (GenScript, pLentiCRISPR v2) were used to homozygously delete the *TP53* gene in H1975 cells as per the manufacturer’s instructions.

### Cell viability assays

For cell viability assays using WST-1 (Sigma-Aldrich, 11644807001) or 3-(4,5-dimethylthiazol-2-yl)-2,5-diphenyltetrazolium bromide (MTT; Thermo Fisher Scientific, AC158992500), cells were seeded at a density of 5 × 10^3^ cells/well in 96-well plates in 100 µL of RPMI media and treated with the indicated concentrations of PI alone for 72 hours or in combination with navitoclax for 96 hours. WST-1 reactions were analyzed by measuring absorbance at 450 nm using a microplate reader (Promega), whereas MTT reactions were analyzed by measuring absorbance at 570 nm using a CLARIOstar plate reader (BMG Labtech). For crystal violet analysis of cell viability, cells were first stained with 0.5% (w/v) crystal violet, followed by extraction of stain using 10% glacial acetic acid and measurement of the absorbance of extracted crystal violet stain at 590 nm using a BioTek Synergy H1 plate reader (Agilent Technologies).

IC_50_ values were determined using GraphPad Prism v8.1 software (GraphPad Software; RRID: SCR_002798). Drug synergy was determined using the Bliss independence model as previously described (19).

### Proteasome activity assays

Cells were seeded at a density of 2.5 × 10^4^ cells/well in a 96-well plate for all assays. For the 20S proteasome activity assay, cells were incubated in proteosome loading solution (Sigma-Aldrich, MAK172) for 1 hour at 37°C, followed by measurement of mean fluorescence intensity at Ex/Em = 490/525 nm using a CLARIOstar plate reader. 26S proteasome activity was measured using a luminescence-based assay (Promega Proteasome-Glo, G8622) using a CLARIOstar plate reader.

### Glutathione assay

Levels of reduced glutathione (GSH) in cell lysates were determined using a GSH assay kit (Cayman Chemical, 703002) per the manufacturer’s instructions. Absorbance of the assay reaction mix was measured at 412 nm using a CLARIOstar plate reader.

### ROS assays

Cells were seeded at a density of 2.5 × 10^4^ cells/well in 96-well plates, and following adherence, ROS indicator dye (DCFDA/H2DCFDA - Cellular ROS Assay Kit, Abcam, ab113851) was prepared and added to each well per the manufacturer’s instructions. After incubation for 30 minutes, fluorescence intensity at Ex/Em = 485/535 nm was determined using a CLARIOstar plate reader.

For flow cytometric ROS assay, approximately 1.0 × 10^6^ cells were incubated with ROS indicator dye CM-H2DCFDA (Thermo Fisher Scientific, C6827) for 30 minutes to 1 hour at room temperature according to the manufacturer’s instructions. The H2DCFDA-stained single-cell suspension was analyzed in a BD FACSCanto II flow cytometer (BD Biosciences; USC Norris Flow Cytometry Core) at Ex/Em = 492 to 495/517 to 527 nm. Percentages of ROS-positive single cells (detected by the FITC channel) were recorded using BD FACSDiva 8.0 software (BD Biosciences; RRID: SCR_001456).

### Annexin V–FITC

Cells were seeded at a density of 5.0 × 10^5^ cells in 60-mm dishes and were treated with vehicle (DMSO), 5 nmol/L BTZ, 1 µmol/L QVD-O-Ph, or the combination for 48 hours. Following treatment, cells were harvested, washed with PBS, and resuspended with 100 µL of 1× annexin V binding buffer (BD Biosciences 556454), followed by the addition of annexin V–FITC (BioLegend, 640945) and propidium iodide (Thermo Fisher Scientific, P3566). Cells were then incubated in the dark for 15 minutes at room temperature, and 400 µL of 1× binding buffer was added to the suspension. Cells were analyzed using a FACSCanto flow cytometer (BD Biosciences) at the VCU Flow Cytometry Core and double-negative, annexin V–positive, propidium iodide–positive, and double-positive cell populations quantified using FlowJo software version 10.8.1 (BD Biosciences; RRID: SCR_008520).

### Immunoblot analyses

Cells were seeded at a density of 1.0 × 10^6^ in 10-cm dishes and treated with vehicle (DMSO), 5 nmol/L BTZ, 1 µmol/L QVD-O-Ph, 1 mmol/L NAC, 1 mmol/L GSH-EE, or indicated combinations for 48 hours. Whole-cell lysates were prepared in lysis buffer (20 mmol/L tris (pH 7.4), 137 mmol/L NaCl, 1 mmol/L dithiothreitol, and 1% (3-[(3-cholamidopropyl) dimethylammonio]-1-propanesulfonate). Equal amounts of protein were loaded into an SDS-PAGE, transferred to a nitrocellulose membrane, incubated with antibodies of interest, and analyzed with ECL2 Western Blotting Substrate (Thermo Fisher Scientific, 32132). Primary antibodies were used at a 1:1,000 dilution for p53 (Santa Cruz Biotechnology, DO-1; RRID: AB_628082), GAPDH (Cell Signaling Technology, D16E11; RRID: AB_10622025), cleaved caspase-3 (Cell Signaling Technology, 5A1E; RRID: AB_10831820), NOXA (Invitrogen, MA1-41000; RRID: AB_1077295), ATF3 (Abcam, ab207434; RRID: AB_2734728), and NRF2 (Cell Signaling Technology, D1Z9C; RRID: AB_2715528). Secondary antibodies were used at a 1:2,000 dilution for horseradish peroxidase–linked anti–rabbit IgG (Cell Signaling Technology, #7074; RRID: AB_2099233) and horseradish peroxidase–linked anti–mouse IgG (Cell Signaling Technology, #7076; RRID: AB_330924).

### qRT-PCR

Cells were seeded at a density of 1.0 × 10^6^ in 10-cm dishes and treated with vehicle (DMSO), 5 nmol/L BTZ, 1 mmol/L NAC, 1 mmol/L GSH-EE, or indicated combinations for 48 hours. Cells were harvested and total RNA extracted using Quick-RNA Miniprep Kit (Zymo, R1054) following the manufacturer’s instructions. cDNA was synthesized using Applied Biosystems High Capacity cDNA Reverse Transcription Kit (Thermo Fisher Scientific, 4368814) based on the manufacturer’s protocol. cDNA was amplified in triplicate using PowerTrack SYBR Green Master Mix (Thermo Fisher Scientific, A46109) in the Applied Biosystems StepOnePlus RT-PCR system (Thermo Fisher Scientific). qRT-PCR primers were synthesized with the sequences listed in Supplementary Table S1, and mRNA expression was determined using ΔΔCt.

### Immunofluorescence

Cells were seeded at a density of 5.0 × 10^2^ cells/well in 4-well culture slides and treated with 10 nmol/L BTZ, 1 mmol/L NAC, or the combination for 24 hours. Following treatment, cells were fixed by incubating with 4% paraformaldehyde for 30 minutes at room temperature. After a 2-minute wash with 1× PBS, cells were permeabilized and blocked using 0.2% Triton-X in 5% BSA for 30 minutes at room temperature. Following a wash with 1× PBS, cells were incubated with primary antibody diluted in blocking buffer for 1 hour at room temperature. After washing three times with 1× PBS, cells were incubated with secondary antibody for 1 hour at room temperature. After washing three times with 1× PBS, slides were cover-slipped using DAPI with mounting media. Fluorescent cells were imaged using either a Leica fluorescent microscope at 20× magnification (Leica) or an LSM 880 Airyscan Confocal Microscope (Carl Zeiss Microscopy) at 40× magnification. NRF2 primary antibody was used at 1:100 dilution (Santa Cruz Biotechnology, sc-13032; RRID: AB_2263168). Mouse anti–rabbit IgG-CFL 594 secondary antibody (Santa Cruz Biotechnology, sc-516250) was used at 1:300 dilution.

### *In vivo* studies

All animal studies were conducted in accordance with VCU Institutional Animal Care and Use Committee guidelines and approval. For xenograft experiments, H1975 (3.0 × 10^6^) or H460 (1.0 × 10^6^) cells were subcutaneously injected into the flank of 6-week-old NOD-SCID-II.2gamma receptor null mice obtained from the VCU Cancer Mouse Models Core. Once tumors became palpable (∼100 mm^3^), mice (*N* = 6/group) were treated for 3 weeks with vehicle (control) or the combination of navitoclax (80 mg/kg) via oral gavage 3×/week, BTZ (1.0 mg/kg) via intraperitoneal injection 2×/week, and carboplatin (25 mg/kg) intraperitoneally once a week. Tumor volumes were measured twice weekly and were calculated as *V* = ½ × *AB*^2^, in which *A* is the longest dimension of the tumor and *B* is the dimension of the tumor perpendicular to *A* as measured using a caliper.

### IHC

Tumors were fixed in 10% formalin phosphate buffer and embedded in paraffin. Tissue embedding and sectioning were performed by the VCU Cancer Mouse Models Core. Slides were blocked for 20 minutes in PowerVision Universal IHC Blocking Diluent (Leica, PV6123) and then stained with cleaved caspase-3 antibody (Cell Signaling Technology, #9664; RRID: AB_2070042), at a 1:500 dilution for 45 minutes. Staining was performed using a Leica Bond RX autostainer (Leica) using the Polymer detection system. Images were taken on the Vectra Polaris Automated Quantitative Pathology Imaging System (Akoya Biosciences) at 20× magnification.

### Circulating neutrophil/platelet analysis

After completion of treatment of H1975-xenografted mice (day 22), blood samples (∼0.2 mL) were collected by cheek bleeding using EDTA-coated syringes and immediately analyzed for neutrophil and platelet counts using a Hemavet 950FS (Drew Scientific) in the VCU Cancer Mouse Models Core.

### Statistical analyses

All quantitative data are shown as ± 1.0 SD from at least three independent experiments, each of which included technical duplicates. All pairwise statistical comparison *t* tests were performed using GraphPad Prism software version 8.1 or Microsoft Excel. *P* ≤ 0.05 was considered statistically significant.

### Data availability

The data generated in this study are available upon request from the corresponding authors.

## Results

### NSCLC cells expressing Onc-p53 exhibit enhanced proteasome activity and sensitivity PIs

**Figure 1:**
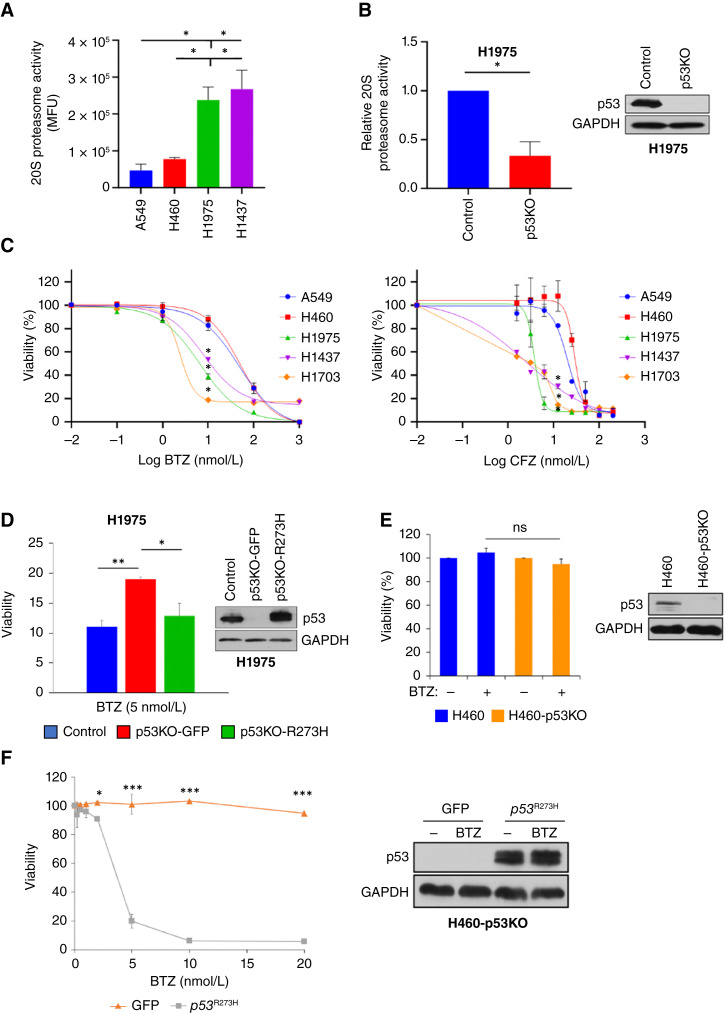
Onc-p53 regulates basal levels of 20S proteasome activity and is required for BTZ-induced cytotoxicity in NSCLC cells. **A,** 20S proteasome activity was determined in the indicated cell lines using a fluorometric proteosome activity assay and plotted as mean fluorescence units (MFU). All pairwise comparisons were made using the Student *t* test. **B,** (Left) Relative 20S proteasome activity in lysates of H1975-Control vs. H1975-p53KO cells. (Right) Cell lysates of H1975-Control and H1975-p53KO (p53KO) cell lines generated using CRISPR/Cas9 were immunoblotted with p53 and GAPDH antibodies. **C,** The indicated cells were treated with vehicle or increasing concentrations of BTZ (left) or CFZ (right) for 72 hours, and cell viability was determined by the 3-(4,5-dimethylthiazol-2-yl)-2,5-diphenyltetrazolium bromide (MTT) assay. A paired *t* test was performed to analyze differences in viability among the cell lines. **D,** (Left) H1975-Control or H1975-p53KO cells expressing GFP or p53^R273H^ were treated with BTZ (5 nmol/L) for 48 hours, and cell viability was determined by trypan blue exclusion assay. (Right) Lysates of the indicated cell lines were immunoblotted with p53 and GAPDH antibodies. **E,** (Left) H460 or H460-p53KO (18) cells were treated with vehicle (−) or BTZ (5 nmol/L) for 72 hours, and cell viability was determined by WST-1 assay. (Right) Cell lysates of H460 and H460-p53KO cells were immunoblotted with p53 and GAPDH antibodies. **F,** (Left) H460-p53KO cells stably expressing GFP or p53^R273H^ were treated with increasing concentrations of BTZ for 72 hours, and cell viability was determined by WST-1 assay. (Right) Cell lysates of H460-p53KO cells stably expressing GFP or p53^R273H^ were immunoblotted with p53 and GAPDH antibodies. *, *P* < 0.05; **, *P* < 0.01; ***, *P* < 0.005; ns, *P* > 0.05. Error bars indicate ± 1.0 SD.

Based on findings in multiple other solid tumors that Onc-p53 elevates proteasome subunit gene expression and activity (11), we hypothesized that NSCLC cells expressing Onc-p53 might also demonstrate similar upregulation of proteasome activity and as a result, also exhibit enhanced cytotoxic sensitivity toward PIs. Therefore, we first assayed 20S proteasome activity in a panel of NSCLC cell lines with differing p53 mutational status (Fig. 1A). As seen in other solid tumor cell types (11), Onc-p53–expressing cells demonstrated three- to fourfold higher 20S proteasome activity than WT p53–expressing cells (*P* < 0.05; Fig. 1A). To determine if *Onc-p53* was necessary for the upregulation of proteasome activity, we developed H1975-Control and homozygous *p53* knockout (H1975-p53KO) cells using CRISPR/Cas9 technology [Fig. 1B (right)]. As has been observed in breast cancer cells (11), 20S proteasome activity was strongly suppressed when *Onc-p53* was deleted [Fig. 1B (left)]. These results indicate that Onc-p53 is required for the increased 20S proteasome activity found in *Onc-p53* NSCLC cells.

As the increased 20S proteasome activity in *Onc-p53* NSCLC cells might signal enhanced sensitivity to PIs, we explored PI cytotoxicity in *Onc-p53* versus *WT p53* NSCLC cells. Indeed, H1975 (*p53*^R273H^), H1437 (*p53*^R267P^), and H1703 (*p53*^G262fs^) *Onc-p53* cells were significantly more sensitive to proteasome inhibition by BTZ (IC_50_ values: 7, 8, and 2 nmol/L, respectively) than NSCLC cell lines harboring *WT p53*, including A549 (IC_50_: 49 nmol/L) and H460 [IC_50_: 51 nmol/L; *P* < 0.05; Fig. 1C (left); Table 1]. This differential sensitivity to proteasome inhibition between *Onc-p53* and *WT p53* cells was also manifested using another FDA-approved PI, CFZ [*P* < 0.05; Fig. 1C (right); Table 1].

**Table 1:** IC_50_ values for BTZ and CFZ in indicated NSCLC cell lines

Cell line	*p53* allele	BTZ IC_50_ (nmol/L)	CFZ IC_50_ (nmol/L)
A549	WT	49	20
H460	WT	51	29
H1975	R273H	7	4
H1437	R267P	8	2
H1703	G262*fs*	2	2

p53 alleles in each cell line are noted. *fs* indicates frameshift due to splice donor mutation.

To determine if *Onc-p53* was necessary for the enhanced sensitivity of *Onc-p53* cells to BTZ-induced cell death, we compared the BTZ sensitivity of H1975-Control, H1975-p53KO cells expressing control (GFP) cDNA, and H1975-p53KO cells with rescue expression of a *p53*^R273H^ cDNA (Fig. 1D). Consistent with loss of p53 specifically driving the effect of the CRISPR p53 knockout on BTZ sensitivity, we observed that H1975-p53KO-GFP cells were significantly less sensitive to 5 nmol/L BTZ than H1975-Control cells (20% vs. 10% viable after 48 hours; *P* < 0.01; Fig. 1D), whereas p53^R273H^ expression restored sensitivity to BTZ (12% viability at 48 hours; *P* < 0.05 for comparison with p53KO-GFP; Fig. 1D).

In contrast, H460 cells with CRISPR knockout of the *WT p53* gene [H460-p53KO; Fig. 1E (right); ref. 18] did not show sensitization to BTZ treatment relative to parental H460 cells [<5% loss of viability in both cell lines; Fig. 1E (left)]. However, expression of exogenous *p53*^R273H^ cDNA versus GFP control in H460-KO cells [Fig. 1F (right)] robustly conferred sensitivity to BTZ cytotoxicity [IC_50_: 4 nmol/L; Fig. 1F (left)]. In alignment with *p53*^R273H^ conferring BTZ sensitivity to H460-p53KO cells, proteasome activity was also significantly induced in H460-p53KO cells expressing *p53*^R273H^ versus *GFP*, whether 20S or 26S proteasome activity was assayed (∼2- and 10-fold induction, respectively; *P* < 0.05 for both; Supplementary Fig. S1A–S1C). These findings suggest that *Onc-p53* induces proteasome activity and renders NSCLC cells differentially more vulnerable to PI treatment.

### Onc-p53 attenuates GSH levels in NSCLC cells

**Figure 2:**
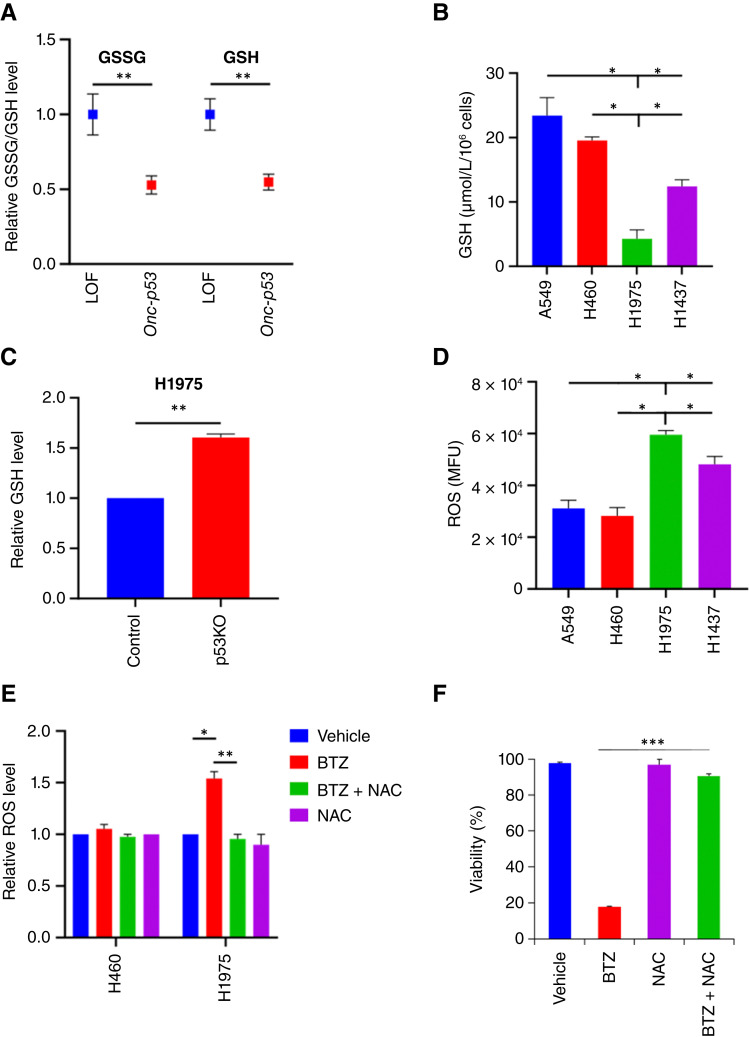
*Onc-p53* NSCLC cells exhibit basal and PI-induced oxidative stress. **A,** Average oxidized (GSSG) vs. reduced (GSH) levels of GSH obtained from metabolomic profiles (DepMap) of NSCLC cell lines that either express Onc-p53 protein (Onc-p53) or lack p53 protein expression due to homozygous deletion/frameshift/premature termination mutations (loss of function; LOF). Data were analyzed using the unpaired *t* test. **B,** GSH concentration was determined in lysates of the indicated cell lines. **C,** Relative GSH concentrations were determined in lysates of H1975-Control vs. H1975-p53KO cells. **D,** Levels of ROS were determined in the indicated cell lines using a fluorescent ROS detection assay and plotted as mean fluorescence units (MFU). **E,** H460 or H1975 cells were treated with vehicle, BTZ (5 nmol/L), NAC (1 mmol/L), or BTZ + NAC for 24 hours. Relative ROS levels normalized to vehicle treatment for each cell line were determined by staining with DCFH-DA followed by flow cytometry. **F,** H1975 cells were treated as per **E** for 48 hours, and cell viability was assessed by trypan blue exclusion. All pairwise comparisons were made using the Student *t* test. *, *P* < 0.05; **, *P* < 0.01; ***, *P* < 0.005. Error bars indicate ± 1.0 SD.

Considering that the mechanism of BTZ cytotoxicity often involves induction of ROS in sensitive cancer cells (17) and that Onc-p53 might alter the tumor cell oxidative state (15), we mined the metabolomic profiles of 52 human NSCLC cell lines that included those expressing *Onc-p53* alleles versus those exhibiting *p53* loss-of-function (homozygous deletion, frameshift, and early termination alleles associated with loss of heterozygosity) for abundance of the key antioxidant metabolite GSH [both reduced (GSH) and oxidized species (GSSH) of GSH; ref. 20]. The average GSH/GSSG levels were ∼50% lower in *Onc-p53* NSCLC cell lines (*n* = 41) compared with the loss-of-function *p53* cell lines (*n* = 11; *P* < 0.01; Fig. 2A). We then measured total GSH levels in the *WT* and *Onc-p53* NSCLC cell lines utilized in Fig. 1A, and observed that the average levels of GSH in *Onc-p53*–expressing cell lines were significantly lower than those in *WT p53* cell lines (*P* < 0.05; Fig. 2B). Furthermore, loss of *Onc-p53* in H1975-p53KO versus H1975-Control cells led to a 70% increase in GSH levels (*P* < 0.01; Fig. 2C). Thus, *Onc-p5*3 expression is associated with GSH depletion in NSCLC cells.

### Induction of ROS by BTZ requires Onc-p53

Based on the depletion of GSH levels seen with *Onc-p53* expression, we inferred that the presence of *Onc-p53* might lead to higher levels of basal ROS as well as potentially toxic accumulation of ROS upon exposure to BTZ. Indeed, H1975 and H1437 *Onc-p53* cells demonstrated significantly higher average levels of basal ROS than H460 and A549 *WT p53* cells as assessed by fluorescent ROS indicator dye (∼2-fold increase, *P* < 0.05; Fig. 2D). To determine if Onc-p53 can solely raise basal ROS levels in isogenic NSCLC cells, we observed ∼4-fold induction of basal ROS levels in H460-p53KO cells expressing p53^R273H^ versus GFP (Supplementary Fig. S1D). We then explored whether BTZ further modulates the intracellular ROS level in *WT* versus *Onc-p53* cells by staining BTZ-treated or BTZ + ROS scavenger NAC–treated H1975 versus H460 cells with ROS indicator dye followed by flow cytometry. Interestingly, BTZ significantly increased ROS levels in *Onc-p53* H1975 cells (*P* < 0.05) but not in *WT p53* H460 cells, and inclusion of NAC reversed the increase in ROS in H1975 cells (*P* < 0.01; Fig. 2E). Furthermore, NAC or the GSH mimetic GSH-EE abrogated BTZ-induced cell death in both H1975 (*P* < 0.005 and *P* < 0.05 for NAC and GSH-EE, respectively; Fig. 2F; Supplementary Fig. S2A) and H1703 (*P* < 0.005; Supplementary Fig. S2B) cells, suggesting that levels of cellular ROS scavengers, such as GSH, were relatively deficient in BTZ-treated *Onc-p53* cells, allowing accumulation of toxic levels of ROS. Taken together, these results suggest that the BTZ sensitivity of *Onc-p53* NSCLC cells is due to an induction of toxic levels of ROS not seen in *WT p53* NSCLC cells.

### BTZ induces NOXA and caspase-dependent apoptosis

**Figure 3:**
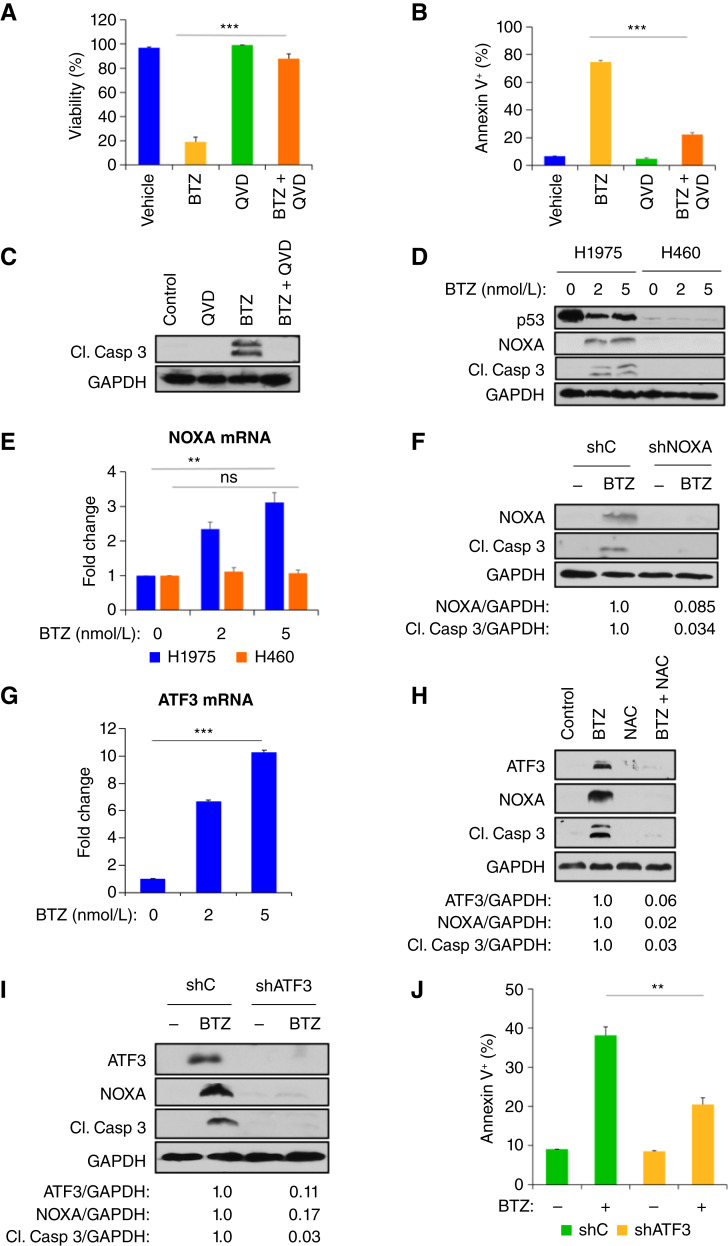
BTZ induces ATF3/NOXA and caspase-dependent apoptosis. **A,** H1975 cells were pretreated (30 minutes) with vehicle or the pan-caspase inhibitor QVD-OPh (QVD; 1 µmol/L) and then treated with vehicle or BTZ (5 nmol/L) for 48 hours. Cell viability was determined by trypan blue exclusion assay. **B,** H1975 cells were treated as in **A**, and the percentage of apoptotic cells was determined by annexin V–propidium iodide staining followed by FACS analysis. **C,** Cell lysates from **A** were immunoblotted with the indicated antibodies. **D,** H1975 or H460 cells were treated with vehicle or 2 or 5 nmol/L of BTZ for 48 hours, and cell lysates were immunoblotted with the indicated antibodies. **E,** RNA from **D** was extracted and subjected to qRT-PCR with *NOXA* primers. **F,** H1975 cells stably expressing shC or shNOXA were treated with vehicle or BTZ (5 nmol/L) for 48 hours, and cell lysates were immunoblotted with the indicated antibodies. **G,** H1975 cells were treated with vehicle or 2 or 5 nmol/L of BTZ for 48 hours, and mRNA expression of ATF3 was analyzed by qRT-PCR. **H,** H1975 cells were treated with vehicle, NAC (1 mmol/L), BTZ (5 nmol/L), or BTZ + NAC for 48 hours, and cell lysates were immunoblotted with indicated antibodies. **I,** H1975 cells stably expressing control shRNA (shC) or ATF3 shRNA (shATF3) were treated with vehicle or BTZ (5 nmol/L) for 48 hours, and cell lysates were immunoblotted with the indicated antibodies. **J,** H1975 cells stably expressing shC or shATF3 were treated with vehicle or BTZ (5 nmol/L) for 48 hours, and the percentage of apoptotic cells was determined by annexin V–propidium iodide staining followed by FACS analysis. **, *P* < 0.01; ***, *P* < 0.005; ns indicates *P* > 0.05. Error bars indicate ± 1.0 SD. Cl. Casp 3, cleaved caspase-3.

To determine the mode of action of BTZ-induced cell death in *Onc-p53*–expressing NSCLC cells, we first determined the contribution of apoptosis by testing the effect of the pan-caspase inhibitor QVD-OPh on BTZ-induced cell death of H1975 cells, and remarkably, cell death was almost completely rescued by QVD-OPh (*P* < 0.005; Fig. 3A). BTZ also induced cell surface exposure of the apoptotic marker annexin V in nearly 80% of cells at 48 hours as measured by flow cytometry, which was partially rescued by QVD-OPh (*P* < 0.005; Fig. 3B). Moreover, the apoptosis marker cleaved caspase-3 was induced by BTZ in H1975 cells, with complete rescue of caspase-3 cleavage by QVD-OPh (Fig. 3C). Taken together, these data strongly suggest that BTZ-induced cell death is largely mediated by apoptosis.

Given that the mechanism of BTZ-induced cell death of *Onc-p53* H1975 cells is apoptotic, as is also seen in multiple myeloma cells (21), we therefore investigated whether the expression of the BH3-only proapoptotic protein NOXA, which is critical for BTZ-induced apoptosis in multiple myeloma cells (21), was also induced in NSCLC cells. Indeed, NOXA protein expression was strongly induced after BTZ exposure in both H1975 and H1437 *Onc-p53* cells but not H460 *WT p53* cells (Fig. 3D; Supplementary Fig. S2C). Furthermore, the induction of NOXA protein in H1975, but not H460, cells was mirrored by *NOXA* mRNA expression, suggesting that BTZ regulates NOXA primarily via transcription (Fig. 3E).

To explore the requirement of NOXA for BTZ-induced apoptosis in H1975 cells, we stably expressed scrambled shRNA or two independent shNOXA constructs in H1975 and H1437 cells and exposed both cell lines to vehicle or BTZ (Fig. 3F; Supplementary Fig. S2D, S2E). Whereas NOXA and cleaved caspase-3 were induced by BTZ in either cell line expressing control shRNA, silencing of NOXA resulted in significant reduction of cleaved caspase-3 levels after BTZ treatment (Fig. 3F; Supplementary Fig. S2D, S2E), reflective of a requirement for NOXA for BTZ-induced apoptosis in *Onc-p53*–expressing H1975 and H1437 cells.

### ATF3 mediates oxidative stress–dependent induction of NOXA by BTZ in *Onc-p53* NSCLC cells

As NOXA induction by BTZ occurred at the level of mRNA expression, we next investigated possible factor(s) controlling *NOXA* transcription in the setting of BTZ exposure. Though *NOXA* can be transcriptionally regulated by numerous transcription factors, ATF3 and ATF4 are both known to regulate *NOXA* in the setting of cellular stress (22), and we noted that transcription of *ATF3* mRNA was strongly induced in BTZ versus vehicle-treated H1975 cells (Fig. 3G), whereas *ATF4* mRNA levels did not change (Supplementary Fig. S3A). Analysis of ATF3 protein expression likewise revealed strong induction, along with NOXA and cleaved caspase-3, upon BTZ treatment of H1975 or H1437 cells (Fig. 3H; Supplementary Figs. S2C and S3B). Moreover, analysis of ATF3, NOXA, and cleaved caspase-3 protein induction over a 48 hours time course after BTZ exposure revealed peak ATF3 expression at 24 hours, whereas NOXA and cleaved caspase-3 peaked at 48 hours, consistent with ATF3 acting upstream of induction of both NOXA and apoptotic caspase-3 cleavage (Supplementary Fig. S3C).

To better understand the epistatic relationship between ROS accumulation/oxidative stress and BTZ-mediated induction of ATF3, NOXA, and caspase-3 cleavage (as in Fig. 2F; Supplementary Fig. S2A and S2B), we monitored ATF3 and NOXA protein and mRNA levels, as well as cleavage of caspase-3, in H1975 cells treated with BTZ in the presence or absence of NAC or GSH-EE (Fig. 3H; Supplementary Fig. S3D–S3H). Strikingly, NAC and GSH-EE both effectively abrogated BTZ-mediated induction of ATF3, NOXA, and cleaved caspase-3, placing NAC/GSH-EE–sensitive oxidative stress epistatically upstream of ATF3, NOXA and cleaved caspase-3 induction in BTZ-treated *Onc-p53* cells.

We next determined whether ATF3 is required for BTZ induction of NOXA and apoptosis in *Onc-p53* NSCLC cells, by interrogating NOXA and cleaved caspase-3 induction in BTZ-treated H1975 cells expressing ATF3 versus control shRNA. Indeed, relative to H1975 cells stably expressing control shRNA, NOXA and cleaved caspase-3 induction was attenuated upon BTZ treatment in H1975 clones stably expressing two independent shATF3 constructs (Fig. 3I; Supplementary Fig. S3I). Moreover, ATF3 knockdown led to a 50% reduction in the annexin V–positive apoptotic cell population upon BTZ exposure (*P* < 0.01; Fig. 3J). These results suggest that BTZ initiates an oxidative stress–dependent signaling cascade in *Onc-p53* NSCLC cells in which ATF3 transcriptional induction downstream of BTZ exposure induces NOXA expression and ultimately, apoptosis.

### BTZ induces nuclear relocalization and accumulation of NRF2 in *Onc-p53*–expressing NSCLC cells

**Figure 4:**
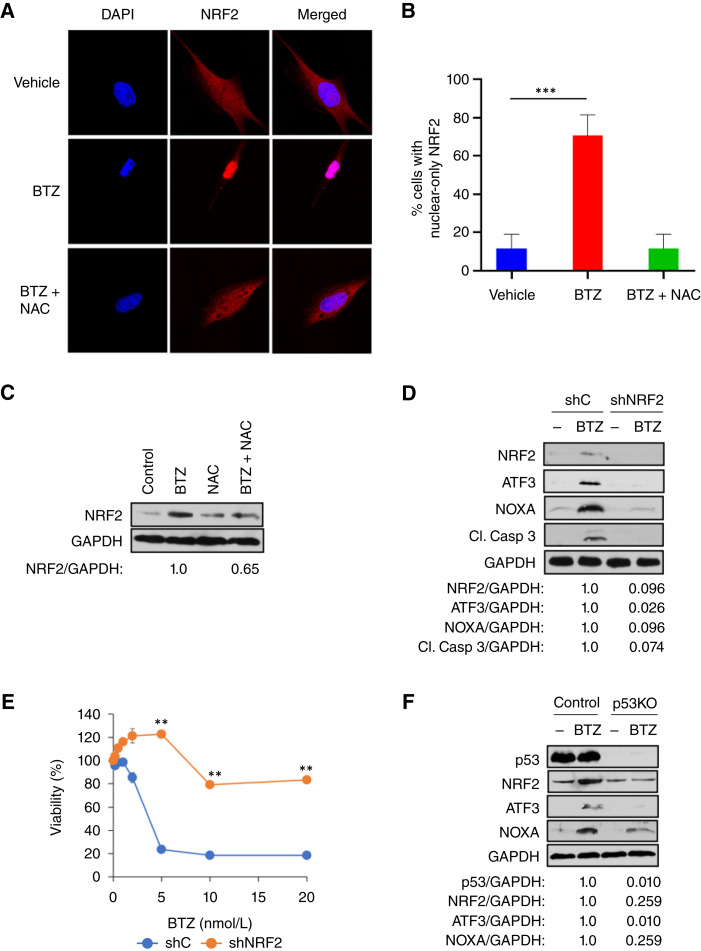
The Onc-p53–NRF2–ATF3–NOXA pathway contributes to BTZ-induced apoptosis in *Onc-p53* NSCLC cells. **A,** H1975 cells were treated with vehicle or BTZ (10 nmol/L) ± NAC (1 mmol/L) for 24 hours, and confocal immunofluorescence microscopy was performed using NRF2 antibody (red) and DAPI to highlight nuclei (blue). Representative individual and merged images (40× magnification) of NRF2 and DAPI stains are shown. **B,** H1975 cells were treated with vehicle, BTZ, or BTZ + NAC, followed by NRF2 and DAPI staining as in **A** and fluorescence microscopy. The percentage of cells with NRF2 localization exclusive to the nucleus was determined from 10 random fields (>10 cells/field counted) at 20× magnification. A pairwise comparison was made using the Student *t* test between the two groups. **C,** H1975 cells were treated with vehicle, NAC (1 mmol/L), BTZ (5 nmol/L), or BTZ + NAC for 48 hours, and cell lysates were immunoblotted with NRF2 and GAPDH antibodies. **D,** H1975 cells stably expressing control shRNA (shC) or NRF2 shRNA (shNRF2) were treated with vehicle or BTZ (5 nmol/L) for 48 hours, and cell lysates were immunoblotted with indicated antibodies. **E,** H1975 shC and shNRF2 cells were treated with vehicle or the indicated concentrations of BTZ for 72 hours, and cell viability was determined by WST-1 assay. **F,** H1975-Control or H1975-p53KO cells were treated with vehicle (−) or BTZ (5 nmol/L) for 48 hours, followed by immunoblotting of cell lysates with indicated antibodies. **, *P* < 0.01; ***, *P* < 0.001. Error bars indicate ± 1.0 SD.

We next addressed the mechanism by which oxidative stress induced by BTZ might be connected to ATF3/NOXA induction. Of potential regulators of ATF3, NRF2 is a well-known transcription factor regulating the cellular antioxidant response, and *ATF3* transcription can be directly regulated by NRF2 (23–25). Under basal unstressed conditions, NRF2 is held in an inactive cytpoplasmic complex with the E3 ubiquitin ligase KEAP1, but upon induction of oxidative cellular stress, NRF2 is released from KEAP1, translocates to the nucleus, and regulates numerous cellular stress–response transcriptional programs (26, 27). We thus examined the subcellular localization of NRF2 in vehicle versus BTZ-treated H1975 cells by immunofluorescence and observed that NRF2 relocalized from a pan-cellular cytosolic and nuclear localization to an exclusively nuclear localization upon BTZ treatment (Fig. 4A and B). Moreover, the nuclear redistribution of NRF2 upon BTZ exposure was abrogated by NAC, indicating that NRF2 relocalization was likely due to oxidative stress (Fig. 4A and B; ref. 27). Furthermore, in parallel to nuclear redistribution, total cellular NRF2 protein abundance was also induced in BTZ versus vehicle-treated H1975 cells, consistent with reported NRF2 stabilization that occurs upon dissociation from KEAP1 (Fig. 4C; ref. 27). NAC treatment reduced the induction of NRF2 abundance consistent with an oxidative stress–dependent mechanism regulating NRF2 abundance after BTZ exposure (Fig. 4C). Taken together, the abrogation of BTZ-mediated nuclear relocalization and stabilization of NRF2 by NAC aligns with the inhibition of BTZ-mediated ATF3/NOXA/cleaved caspase-3 induction and loss of cell viability by NAC and GSH-EE (Fig. 2F and 3H; Supplementary Figs. S2A and S2B, S3D–S3H), suggesting that NRF2 could be the factor connecting BTZ-induced oxidative stress to induction of ATF3/NOXA and apoptosis in *Onc-p53* NSCLC cells.

### NRF2 is required for the induction of ATF3/NOXA and cell death by BTZ in *Onc-p53*–expressing NSCLC cells

To determine whether NRF2 is required upstream of BTZ-mediated cytotoxic signaling via ATF3/NOXA in *Onc-p53* NSCLC cells, we first treated H1975 cells stably expressing NRF2 or control shRNA with vehicle or BTZ and immunoblotted cell lysates for NRF2, ATF3, NOXA, and cleaved caspase-3 (as a surrogate for apoptosis; Fig. 4D). Whereas BTZ treatment of shControl-expressing H1975 cells revealed the expected induction of ATF3/NOXA and cleaved caspase-3, shNRF2 expression strongly attenuated BTZ induction of ATF3/NOXA and caspase-3 cleavage (Fig. 4D). Moreover, shNRF2 expression rendered H1975 cells highly resistant to BTZ-mediated cell death, as only 20% loss of viability was observed at BTZ doses as high as 20 nmol/L, whereas shControl-expressing H1975 cells exhibited expected sensitivity to BTZ with ∼80% loss of viability with exposure to only 5 nmol/L BTZ (*P* < 0.01; Fig. 4E). These results indicate that NRF2 is necessary for BTZ induction of ATF3/NOXA and apoptosis in *Onc-p53* H1975 NSCLC cells.

### Onc-p53 is required for BTZ-mediated induction of NRF2/ATF3/NOXA and apoptosis in *Onc-p53–* expressing NSCLC cells

We next addressed if activation of the proposed NRF2–ATF3–NOXA signaling cascade in BTZ-treated cells ultimately requires ongoing expression of Onc-p53. Indeed, H1975-p53KO cells, which exhibit resistance to BTZ-mediated cytotoxic cell death (Fig. 1D), demonstrated reduced or nearly absent induction of NRF2, ATF3, and NOXA proteins as well as *ATF3* and *NOXA* mRNA (Fig. 4F; Supplementary Fig. S4A and S4B). These results indicate that Onc-p53 is a necessary upstream component of an NRF2–ATF3–NOXA signaling cascade that leads to apoptosis in *Onc-p53* H1975 cells.

### BH3-mimetics sensitize *Onc-p53*–harboring NSCLC cells to BTZ cytotoxicity

**Figure 5:**
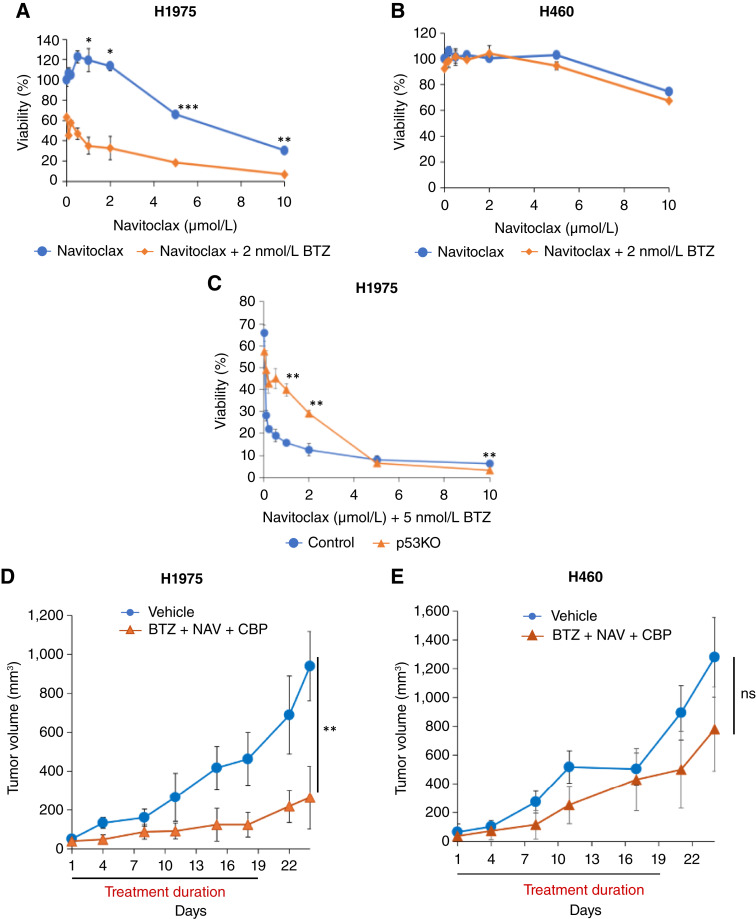
Navitoclax enhances BTZ-induced cytotoxicity. **A** and **B,** H1975 and H460 cells were treated with vehicle or the indicated concentrations of navitoclax with/without BTZ (2 nmol/L) for 96 hours. Cell viability was determined by WST-1 assay. **C,** H1975-Control or H1975-p53KO cells were treated with vehicle or the indicated concentrations of navitoclax with/without BTZ (5 nmol/L) for 96 hours. Cell viability was determined by WST-1 assay. **D** and **E,** NOD-SCID-II.2 receptor gamma null (NSG) mice were injected with 3.0 × 10^6^ H1975 (**D**) or 1.0 × 10^6^ H460 cells (**E**), and once tumors became palpable, mice were treated for 3 weeks with vehicle (control) or the combination of BTZ (1.0 mg/kg) 2×/week via intraperitoneal injection + navitoclax (NAV; 80 mg/kg) 3×/week via oral gavage + carboplatin (CBP; 25 mg/kg) once weekly via intraperitoneal injection. The period of treatment is indicated by the horizontal bar, and tumor volumes were measured twice weekly (*N* = 6 mice/group). *, *P* < 0.05; **, *P* < 0.01; ***, *P* < 0.005; ns indicates *P* > 0.05. Error bars indicate ± 1.0 SD., except in **D** and **E** in which error bars indicate SEM.

To further explore additional mechanisms of specifically enhancing PI cytotoxicity in *Onc-p53* NSCLC cells, we considered whether targeting BCL-2 family antiapoptotic proteins might cooperatively improve BTZ cytotoxicity as well. First, we investigated the basal levels of antiapoptotic BCL-2 family proteins in H1975, H1437, and H1703 *Onc-p53* cells and observed that BCL-X_L_ and MCL-1 were variably expressed among the cell lines, but BCL-2 was undetectable in H1437 cells (Supplementary Fig. S5A). As NOXA specifically binds to and inactivates prosurvival MCL-1 (28), we hypothesized that the BH3-mimetics targeting BCL-X_L_, such as navitoclax (ABT-263/navitoclax, a BCL-2/BCL-X_L_ dual-inhibitor; ref. 29), could enhance BTZ-induced cytotoxicity. Indeed, we found that BTZ strikingly enhanced the cytotoxicity of navitoclax in *Onc-p53* H1975, H1437, and H1703 cells (*P* < 0.05; Fig. 5A; Supplementary Fig. S5B and S5C) but not in *WT p53* H460 cells (Fig. 5B), with a clear synergistic effect noted in H1975 cells as determined by Bliss score (Supplementary Fig. S5D; ref. 19). Furthermore, H1975-p53KO cells were much less sensitive to the combination of BTZ and navitoclax as compared with H1975-Control cells (*P* < 0.01; Fig. 5C), indicating that the combinatorial synergy of BTZ and navitoclax is Onc-p53 dependent.

These striking *in vitro* data suggest that the combination of BTZ and a BH3-mimetic could represent an effective rational therapeutic strategy in NSCLC tumors bearing *Onc-p53* alleles. As such, we tested the *in vivo* efficacy of combining BTZ and navitoclax along with carboplatin (standard-of-care NSCLC chemotherapeutic) in H1975-xenografted NOD-SCID-II.2gamma receptor null mice treated with vehicle or the combination, over 3 weeks. Relative to vehicle-treated tumors, the BTZ/navitoclax/carboplatin combination caused a robust and significant ∼75% reduction in tumor volume after 3 weeks of treatment (*P* < 0.01; Fig. 5D). In contrast, H460 xenografts did not show significant benefit from the combination treatment (Fig. 5E).

Consistent with the proposed apoptotic mechanism of BTZ cytotoxicity in NSCLC cells, IHC staining of the treated tumors for cleaved caspase-3 revealed strong staining (20%–40% of nuclei positive) in tumors exposed to BTZ/navitoclax/carboplatin combination treatment versus vehicle (Supplementary Fig. S6A and S6B). Notably, the BTZ/navitoclax/carboplatin combination was well tolerated, despite the known dose-limiting toxicities of navitoclax, including thrombocytopenia, that prevented its further clinical development (30, 31). Indeed, platelet and neutrophil counts obtained at the endpoint of the BTZ/navitoclax/carboplatin experiment, shown in Fig. 5D, remained within the reference range without any loss of mouse body weight (Supplementary Fig. S6C and S6D; 32). These data support further study of PI/BH3-mimetic/chemotherapy combination therapies in patients with *Onc-p53*–expressing NSCLC tumors.

## Discussion

In the current study, we have identified a vulnerability of NSCLC cells to PIs dependent on Onc-p53 expression which is known to drive excess levels of proteasome activity in multiple tumor settings (11). Indeed, we confirmed that *Onc-p53* NSCLC cells also exhibit significantly higher levels of proteasome activity requiring the ongoing expression of Onc-p53 and that PIs exhibit preferential cytotoxicity in *Onc-p53* versus *WT* or cells lacking *p53 in vitro* and *in vivo*. Surprisingly, BTZ cytotoxic effects in Onc-p53 NSCLC cells were rescued completely by NAC, indicating that oxidative stress is a critical driver of BTZ-dependent cytotoxic effects in *Onc-p53* cells. Importantly, we observed oxidative stress–dependent nuclear translocation of NRF2 and transcriptional activation of ATF3, which in turn was required for NOXA induction and apoptosis in *Onc-p53* NSCLC cells treated with BTZ. Validating the translational potential of BTZ in *Onc-p53* NSCLC, BTZ and the BH3-mimetic navitoclax were synergistically cytotoxic in *Onc-p53* but not *WT p53* NSCLC cells *in vitro,* and BTZ effectively limited the growth of *Onc-p53*–expressing NSCLC xenografts when combined with carboplatin and navitoclax *in vivo*.

Although PIs are incorporated into the standard of care for the treatment of multiple myeloma and other hematologic malignancies, their therapeutic utility in NSCLC, or any other solid tumors, has never been established (33). BTZ and CFZ were previously tested as single agents, or in combination with platinum or other chemotherapeutic agents, in multiple phase I/II NSCLC clinical trials (12, 34) with a modest efficacy signal observed in a few of the combination trials (with docetaxel, gemcitabine, or carboplatin), mostly in relapsed/refractory disease (35, 36). However, patient selection criteria for these trials varied and were not biomarker dependent, precluding the ability to draw definitive conclusions (12). Our *in vitro/in vivo* results strongly highlight the need to both select patients based on *Onc-p53* status and employ rational combinations to truly test the clinical utility of PIs in NSCLC.

*TP53* is among the most frequently mutated tumor-suppressor genes in human cancer, with the unique characteristic that p53 proteins expressed from missense mutated or truncated alleles (*Onc-p53*) acquire emergent oncogenic functions (also termed “gain of function” in the literature), facilitating tumor cell growth, proliferation, and apoptotic evasion (37). Published studies have clearly demonstrated the connection between Onc-p53 and increased proteosome activity in several cancers, including breast, pancreatic, ovarian and prostate cancer (11). Furthermore, NRF2 was shown to complex with Onc-p53 and bind to the promoter region of 26S proteasome subunit genes, leading to upregulation of proteosome subunit transcription (11, 38). As Onc-p53 has a high tendency to aggregate in cells (39, 40), it has been hypothesized that proteotoxic stress from Onc-p53 aggregates may induce proteasome activity to maintain cellular homeostasis and manage the proteotoxic load (41). Indeed, the p53^R273H^ protein expressed in H1975 cells has been observed to form intracellular aggregates in breast and ovarian cancer cells using an antibody sensitive to high–molecular weight protein aggregates, though the aggregation status of the p53^R267P^ (H1437 cells) and p53^G262fs^ (H1703 cells) proteins has not yet been reported (40, 42). Most importantly, our work for the first time links proteotoxic stress generated by *Onc-p53* to elevated PI sensitivity, which to date has only been noted in B-cell malignancies such as MM and MCL, in which immunoglobulin synthesis causes substantial and ongoing proteotoxic stress (13).

**Figure 6:**
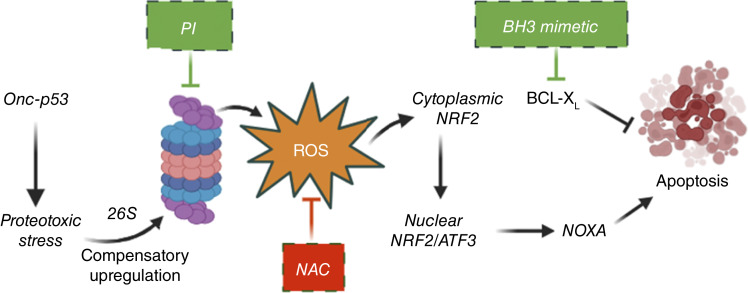
Proposed cytotoxic mechanism of PIs and sensitizing agents in *Onc-p53* NSCLC cells. Onc-p53 induces proteotoxic stress causing compensating upregulation of proteasome activity. PIs further augment proteotoxic stress, leading to ROS accumulation. Toxic ROS accumulation then initiates a cascade including activation and nuclear translocation of NRF2, induction of ATF3 and NOXA, and ultimately, apoptosis, which can be enhanced by BH3 mimetics. NAC is an antioxidant that blocks ROS accumulation, thus blocking nuclear translocation of NRF2, the ATF3/NOXA cascade, and apoptosis. [Created with BioRender.com; Chougoni, K. (2024) BioRender.com/v58l013].

In agreement with published studies, *Onc-p53* NSCLC cells demonstrate higher basal levels of ROS (and lower GSH levels) compared with *WT p53* NSCLC cells (Fig. 2; Supplementary Fig. S1D). Our work, for the first time, also links additional ROS induced by PIs to a cytotoxic cascade that includes ROS-dependent NRF2 activation, downstream activation of ATF3 then NOXA, and subsequently apoptosis (see Fig. 6). Indeed, every step in this cascade can be abrogated by inclusion of the antioxidant NAC or GSH-mimetic GSH-EE during exposure to PI (Figs. 2F, 3H, 4A–C; Supplementary Figs. S2A and S2B, S3D–S3H). What remains unclear is the exact source of both basal and PI-induced ROS. One possible explanation is GSH depletion through means other than consumption by ROS, such as downregulation of transporter or biosynthetic genes regulating the GSH synthesis pathway (43). Another source of ROS could be oxidative stress due to *Onc-p53* itself, either via an induced downstream oxidative stress pathway or through oxidative activity of proteasomes, in which activity is upregulated by Onc-p53 (Figs. 1A, 1B, and 6; Supplementary Fig. S1B and S1C). Further investigation of the underlying mechanism of toxic ROS generation by PIs in *Onc-p53* NSCLC could lead to the identification of novel targets and improved combination therapies that potentiate the selective cancer cell cytotoxicity of PIs in *Onc-p53* NSCLC cells and tumors.

NRF2 is a well-established regulator of antioxidant mechanisms, and its activity is normally suppressed because of ubiquitination and proteasomal degradation caused by cytoplasmic interaction with the E3 ligase KEAP1 (44). Upon cellular exposure to oxidative stress, NRF2 is released from KEAP1 and translocates to the nucleus to activate highly context-specific and cell type–specific transcriptional programs that broadly regulate cellular defenses to oxidation but also impact both oncogenic and tumor-suppressive pathways associated with the hallmarks of cancer (44). Of note, the vast majority of *Onc-p53* tumors and cells maintain wild-type KEAP1 (20), though NRF2 can still be induced in the presence of excess oxidative stress that releases it from KEAP1, both stabilizing NRF2 and allowing it to translocate to the nucleus and activate downstream target genes (45). Notably, we observed accumulation and nuclear relocalization of NRF2 protein in *Onc-p53* cells upon PI treatment, and both of these effects were blocked by NAC or *p53* knockout (Fig. 4A–C, F). Given prior reports in other settings that oxidative stress causes dissociation of the KEAP1/NRF2 complex, we hypothesize that NRF2 accumulation and nuclear relocalization are due to ROS-induced KEAP1/NRF2 dissociation and NRF2 stabilization, as opposed to direct stabilization of NRF2 by PI treatment or induction of NRF2 transcription (45).

Beyond oxidative activation of NRF2, others have also reported that Onc-p53 can form a complex with and coactivate NRF2 as an alternative or additional means of NRF2 activation in *Onc-p53* cancer cells (11, 38). Given the obvious complexity of NRF2 regulation both in normal cells and *Onc-p53* cancer cells, a more detailed understanding of the mechanism of NRF2 regulation after PI treatment of NSCLC cells will require further study beyond the scope of this work.

The induction of NOXA by PIs has been well established in MM, MCL, chronic myelogenous leukemia, and melanoma (17, 46, 47), though the exact mechanism is not entirely clear. In some studies, PIs induced NOXA protein accumulation via protein stabilization through inhibition of the ubiquitin–proteasome pathway (48). In other contexts, however, *NOXA* induction by PIs occurred at the transcriptional level (47). Mechanistically, c-MYC has been reported to mediate PI induction of *NOXA* in melanoma cells (49), whereas *NOXA* transcription can also be regulated by ATF3/ATF4 in the non-PI setting through a variety of signaling pathways, including the endoplasmic reticulum stress pathway (50). In *Onc-p53* NSCLC cells, we observed BTZ-mediated ATF3, but not ATF4, induction (Fig. 3G and H; Supplementary Fig. S3A–S3C), which heretofore has not been observed after PI treatment in other tumor contexts, such as MM (51). Furthermore, downregulation of ATF3 significantly inhibited BTZ induction of NOXA and cell death (Fig. 3I and J). We and others have shown that ATF3 is capable of binding to the *NOXA* promoter in other tumor contexts (e.g., squamous cell carcinoma of the head and neck) after other exposures, such as cisplatin (52, 53). In the current context with BTZ treatment in *Onc-p53* NSCLC cells, we speculate that BTZ-induced ATF3 forms homodimers and binds to the NOXA promoter directly to activate its expression, which we are currently exploring.

Given that BTZ is potently cytotoxic in cultured *Onc-p53* NSCLC cells but seems of limited efficacy as a single agent in unselected patients in clinical trials (12), we hypothesized that a combination of BTZ with an agent that abrogates prosurvival signaling, such as a BH3-mimetic (54, 55), would enhance BTZ efficacy. Specifically, the proapoptotic function of NOXA is to inactivate prosurvival MCL-1, and whereas BCL-X_L_ is robustly expressed in H1975, H1437, and H1703 cells, BCL-2 is expressed in H1975 cells but is undetectable in BTZ-sensitive H1437 cells (Supplementary Fig. S5A), suggesting that a BH3-mimetic that can target BCL-X_L_, such as the dual BCL-2/BCL-X_L_ inhibitor navitoclax, may further enhance cell death in *Onc-p53* H1975, H1437, and H1703 cells already sensitized to BTZ cytotoxicity. Indeed, we observed significant, and for H1975 cells, synergistic, enhancement of cell death after BTZ/navitoclax combination treatment in these cell lines *in vitro* (Fig. 5A; Supplementary Fig. S5B–S5D).

We further demonstrated robust and significant attenuation of tumor growth by a BTZ/navitoclax/carboplatin combination regimen in *Onc-p53* H1975, but not *WT p53* H460, xenografts (Fig. 5D and E), consistent with our hypothesis that PI/BH3 mimetic–based therapy is specifically active in NSCLC tumors bearing *Onc-p53*. The addition of carboplatin chemotherapy to the *in vivo* treatment regimen, as shown in Fig. 5D and E, reflects the common practice in NSCLC clinical trial designs of adding novel agents or rational combinations to standard chemotherapy “backbones” (35, 36). Notably, navitoclax can demonstrate on-target inhibition of BCL-X_L_ in platelets, inducing clinically unacceptable thrombocytopenia (30, 31), but surprisingly, we observed no decrease in platelet counts over the course of the mouse xenograft experiment (Supplementary Fig. S6C).

As the clinical development of navitoclax has been halted because of observed hematologic toxicity (30, 31), next-generation BH3-mimetic drugs in development include WH244, a proteolysis targeting chimera (PROTAC) that is specifically activated to degrade BCL-X_L_ in tumor cells but not in platelets (56), and pelcitoclax, a small-molecule direct BCL-X_L_ inhibitor (57). WH244 is soon to undergo clinical testing but is an optimized version of the BCL-X_L_ PROTAC 753b, which has exhibited an acceptable preclinical safety profile (58), and pelcitoclax is currently undergoing evaluation in clinical trials (e.g., NCT05186012). We envision either of these novel BH3 mimetics replacing navitoclax and serving as a suitable partner for future combination clinical trials with PIs in *Onc-p53* NSCLC.

Taken together, this work demonstrates a novel mechanism of action for PIs in *Onc-p53* NSCLC cells that has not been observed in tumor types in which PIs are currently in clinical use. Our work points the way to potential clinical repurposing of PIs in *Onc-p53* NSCLC and possibly other high-frequency *Onc-p53* tumors (small cell lung cancer, ovarian cancer, and pancreatic cancer, among others). We show enhanced effectiveness of BTZ when combined with carboplatin *in vitro* or in an *in vivo* xenograft model, suggesting that the combination of BTZ with standard-of-care chemotherapy may show effectiveness in selected patients with *Onc-p53* NSCLC. We did not determine if carboplatin enhanced any specific aspect of the BTZ mechanism of action, though it is known that platinum agents generally increase oxidative stress, and thus may drive enhanced NRF2 activation upon BTZ exposure (59).

By carefully mapping the pathway by which PIs kill *Onc-p53* NSCLC cells via proteotoxic stress inducing oxidative stress, followed by the induction of an NRF2–ATF3–NOXA signaling cascade and ultimately apoptosis, we have also developed a rational combination of BTZ with navitoclax as a proof of principle for a future clinical trial involving a PI + next-generation BH3-mimetic combination. Additional translational consideration can be given to PI combinations with agents that target proteotoxic stress [e.g., p97 inhibitors (60)] or agents other than chemotherapy that augment oxidative stress and GSH depletion (61), such as sulfasalazine and APR-246/PRIMA-1 (11, 43, 62, 63).

## Supplementary Material

Table S1 Table S1

Figure S1 Figure S1

Figure S2 Figure S2

Figure S3 Figure S3

Figure S4 Figure S4

Figure S5 Figure S5

Figure S6 Figure S6
