# Supplementary material for: Oncogenic Mutant p53 Sensitizes Non–Small Cell Lung Cancer Cells to Proteasome Inhibition via Oxidative Stress–Dependent Induction of Mitochondrial Apoptosis
Source: Cancer Res Commun. 2024 Oct 15;4(10):2685–98. doi: 10.1158/2767-9764.CRC-23-0637 (PMC11474859; doi:10.1158/2767-9764.CRC-23-0637)
Supplement: Table S1 [file crc-23-0637_table_s1_suppst1.pdf]

Table S1

Gene	Forward Sequence	Reverse Sequence
ATF3	5'- CGC TGG AAT CAG TCA CTG TCA G -3'	5'- CTT GTT TCG GCA CTT TGC AGC TG -3'
ATF4	5'- TTC TCC AGC GAC AAG GCT AAG G -3'	5'- CTC CAA CAT CCA ATC TGT CCC G -3'
NOXA	5'- CTG GAA GTC GAG TGT GCT ACT C -3'	5'- TGA AGG AGT CCC CTC ATG CAA G -3'
p53	5'- AAG GAA ATT TGC GTG TGG AGT -3'	5'- AAA GCT GTT CCG TCC CAG TA -3'
GAPDH	5'- GTC TCC TCT GAC TTC AAC AGC G -3'	5'- ACC ACC CTG TTG CTG TAG CCA A -3'

Table S1. Primer sequences used for qRT-PCR.
