# Supplementary material for: Oncogenic Mutant p53 Sensitizes Non–Small Cell Lung Cancer Cells to Proteasome Inhibition via Oxidative Stress–Dependent Induction of Mitochondrial Apoptosis
Source: Cancer Res Commun. 2024 Oct 15;4(10):2685–98. doi: 10.1158/2767-9764.CRC-23-0637 (PMC11474859; doi:10.1158/2767-9764.CRC-23-0637)
Supplement: Figure S1 [file crc-23-0637_figure_s1_suppsf1.pdf]

Figure S1

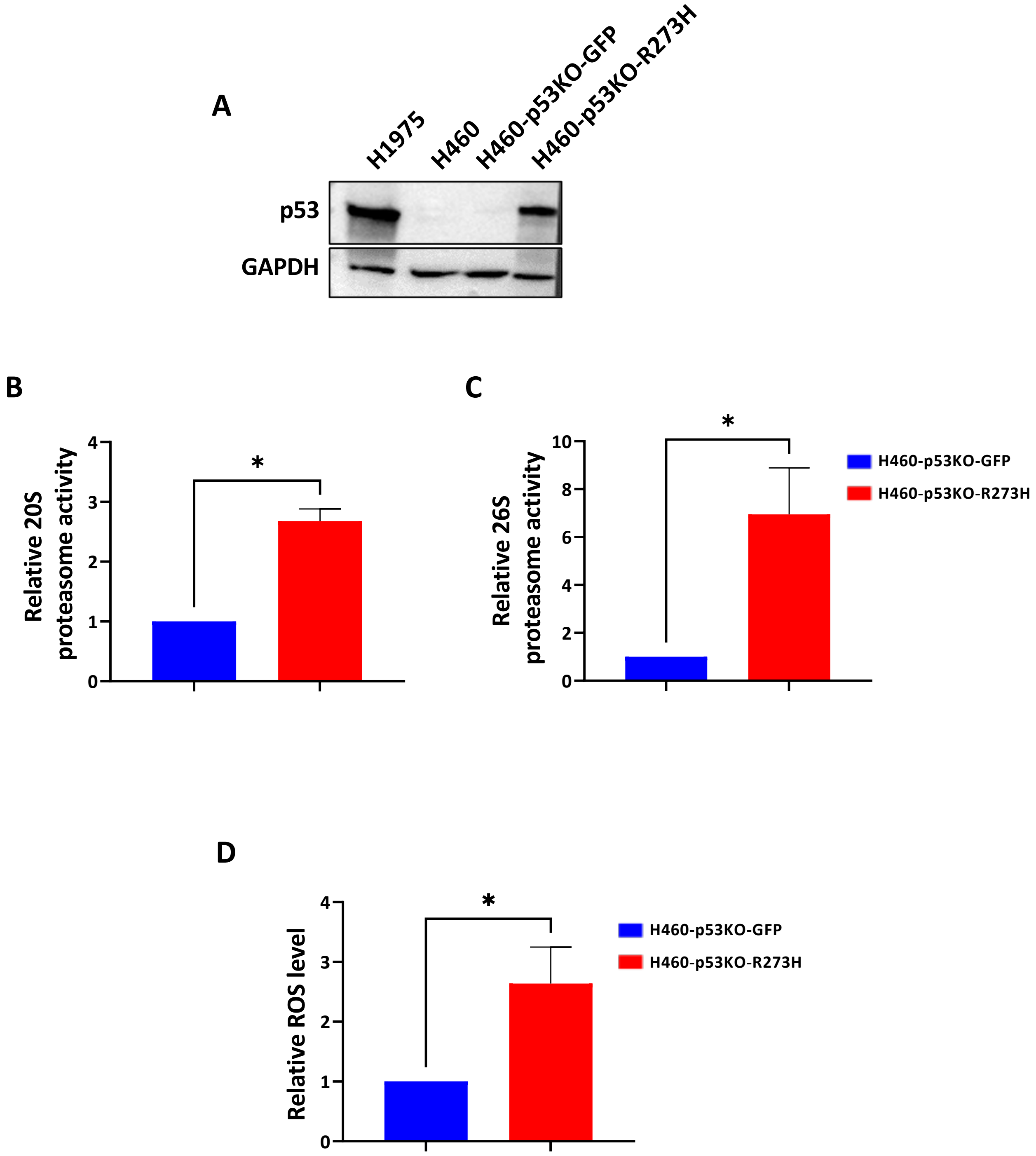

Fig. S1. Isogenic expression of a p53^{R273H} allele in a p53-null NSCLC cell background confers increased basal proteasome activity and ROS levels. A. p53 immunoblot of parental H460, H460-p53KO-GFP and H460-p53KO-R273H cell lines with H1975 cell lysate as a reference for expression level of a native p53^{R273H} allele. GAPDH was a loading control. **B.** Relative 20S proteasome activity was measured using fluorescence-based assay in lysates of H460-p53KO-R273H vs. H460-p53KO-GFP cells. **C.** Relative 26S proteasome activity (Proteasome-Glo, Promega) was measured in lysates of H460-p53KO-R273H vs. H460-p53KO-GFP cells. **D.** Relative ROS level in H460-p53KO-R273H vs. H460-p53KO-GFP cells was determined by fluorescent ROS assay. *p<0.05. Error bars indicate +/- 1.0 S.D.
