# Supplementary material for: Oncogenic Mutant p53 Sensitizes Non–Small Cell Lung Cancer Cells to Proteasome Inhibition via Oxidative Stress–Dependent Induction of Mitochondrial Apoptosis
Source: Cancer Res Commun. 2024 Oct 15;4(10):2685–98. doi: 10.1158/2767-9764.CRC-23-0637 (PMC11474859; doi:10.1158/2767-9764.CRC-23-0637)
Supplement: Figure S2 [file crc-23-0637_figure_s2_suppsf2.pdf]

Figure S2

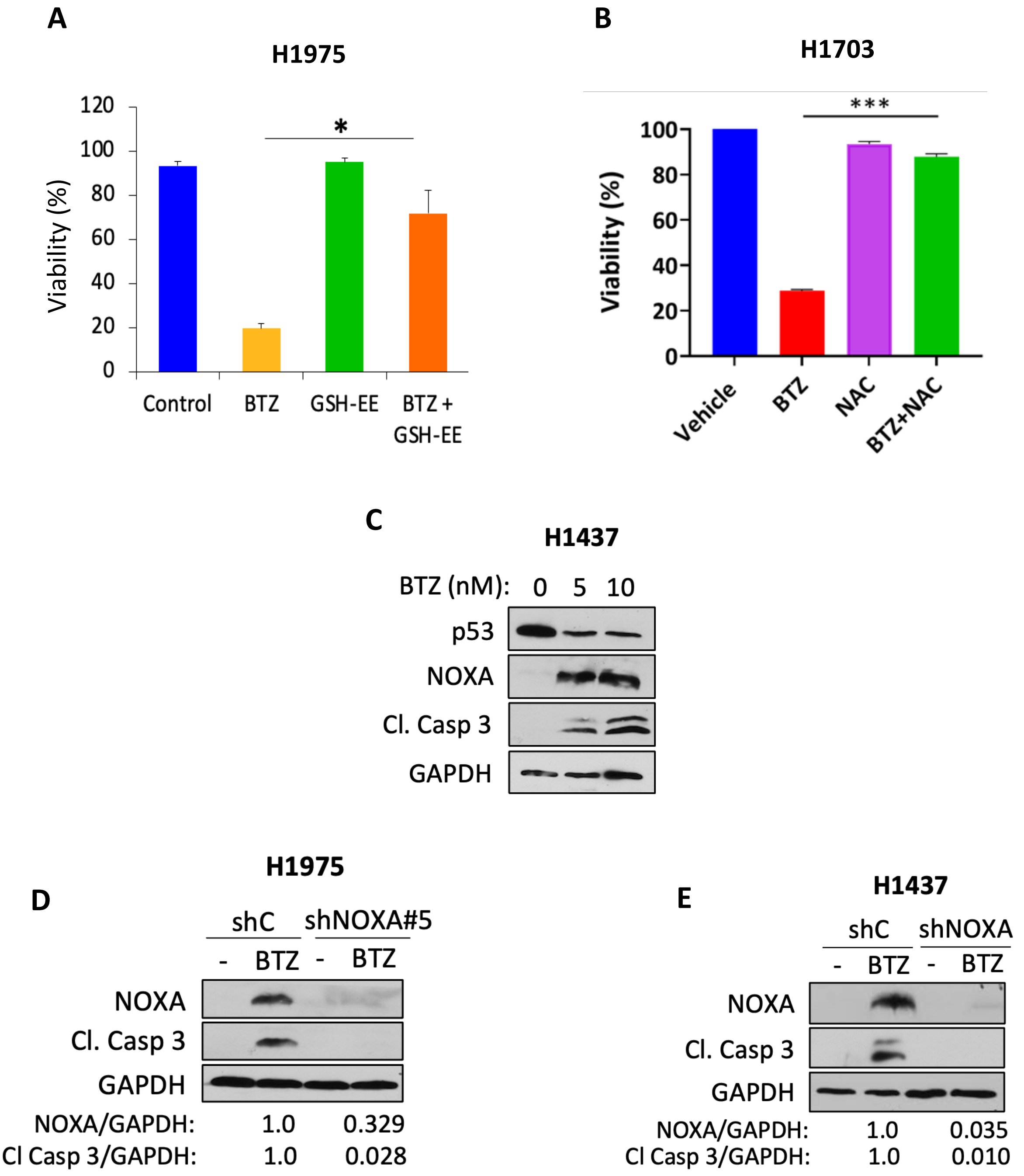

Fig. S2. BTZ-induced cell death depends on ROS and NOXA. A. H1975 cells were treated with vehicle or 5 nM BTZ with or without 1 mM GSH-EE for 48 h. Cell viability was determined by Trypan-blue exclusion assay. **B.** H1703 cells were treated with vehicle or 5 nM BTZ with or without 1 mM NAC for 48 h. Cell viability was determined by crystal violet staining. **C.** H1437 cells were treated with the indicated concentration of BTZ for 48 h. Whole cell lysates were immunoblotted with the indicated antibodies. **D.** H1975 cells stably expressing control shRNA (shC) or an alternate NOXA shRNA, shNOXA#5, were treated with vehicle (-) or BTZ (5 nM) for 48 h and cell lysates were immunoblotted with the indicated antibodies. **E.** H1437 shRNA control and shNOXA cells were treated with and without BTZ (5 nM) for 48 h and cell lysates were immunoblotted with the indicated antibodies. **p*<0.05; ***p<0.001. Error bars indicate +/- 1.0 S.D.
