# Supplementary material for: Oncogenic Mutant p53 Sensitizes Non–Small Cell Lung Cancer Cells to Proteasome Inhibition via Oxidative Stress–Dependent Induction of Mitochondrial Apoptosis
Source: Cancer Res Commun. 2024 Oct 15;4(10):2685–98. doi: 10.1158/2767-9764.CRC-23-0637 (PMC11474859; doi:10.1158/2767-9764.CRC-23-0637)
Supplement: Figure S3 [file crc-23-0637_figure_s3_suppsf3.pdf]

Figure S3

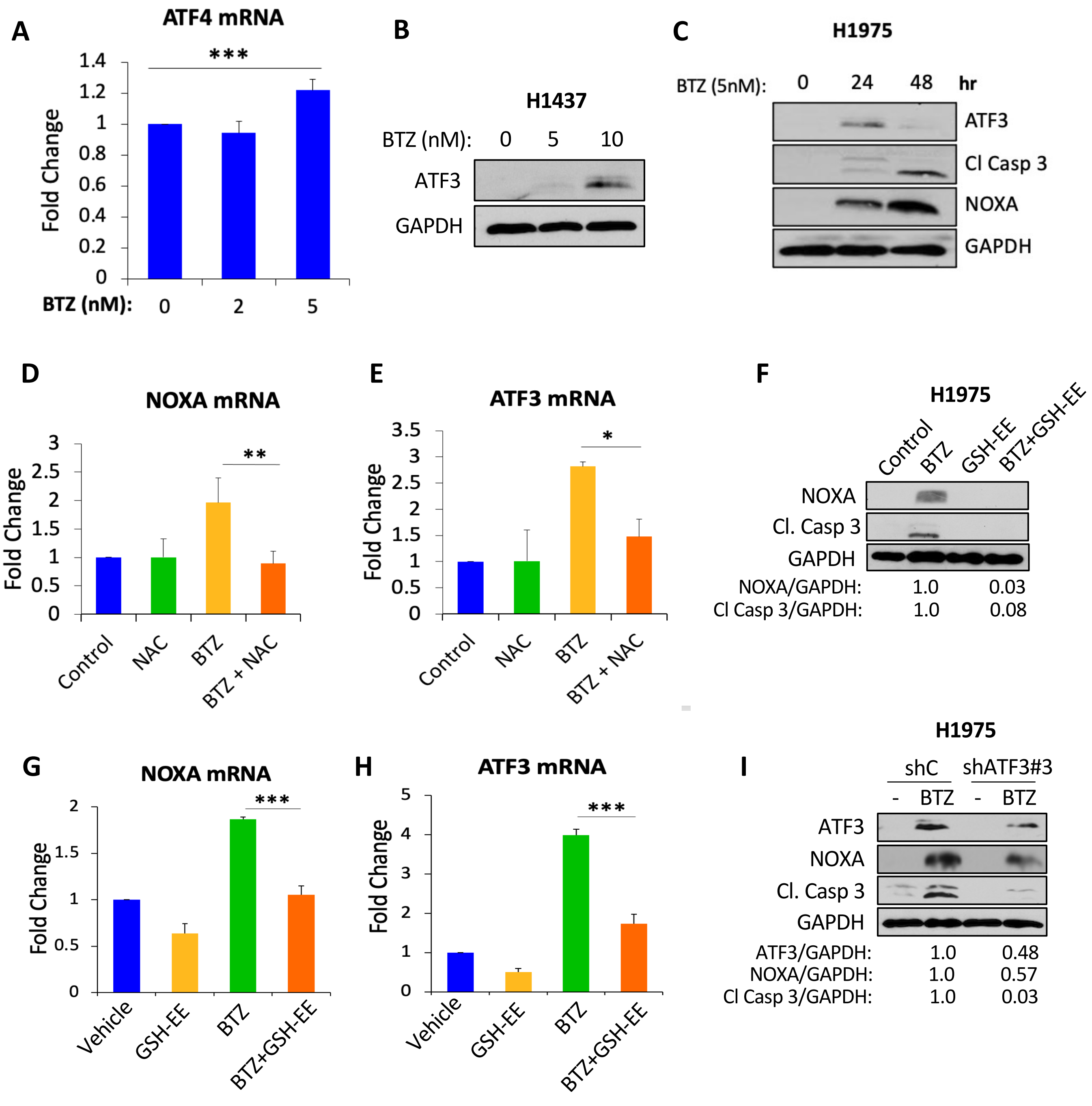

Fig. S3. Oxidative stress-dependent induction of ATF3 upon BTZ exposure in Onc-p53 NSCLC cells drives NOXA induction and cell death. A. H1975 cells were treated with vehicle or BTZ (2, 5 nM) for 48 h and mRNA expression of ATF4 was analyzed by qRT-PCR. **B.** H1437 cells were treated with vehicle, 5, or 10 nM of BTZ for 48 h and cell lysates were immunoblotted with ATF3 and GAPDH antibodies. **C.** H1975 cells were treated with vehicle or BTZ (5 nM) for 24 or 48 h and cell lysates were immunoblotted with indicated antibodies. **D-E.** H1975 cells were treated with vehicle or BTZ (5 nM) with or without NAC (1 mM) or for 48 h and NOXA and ATF3 expression were analyzed by qRT-PCR. **F.** H1975 cells were treated with vehicle or BTZ (5 nM) with or without GSH-EE (1mM; GSH) for 48 h and cell lysates were immunoblotted with indicated antibodies. **G-H.** H1975 cells were treated with vehicle or BTZ (5 nM) with or without GSH-EE (1 mM; GSH) for 48 h and NOXA and ATF3 expression were analyzed by qRT-PCR. **I.** H1975 cells stably expressing control shRNA (shC) or an alternate ATF3 shRNA, shATF3#3, were treated with vehicle (-) or BTZ (5 nM) for 48 h and cell lysates were subjected to immunoblotting with the indicated antibodies. **p*<0.05, ***p*<0.01, ****p*<0.005. Error bars indicate +/- 1.0 S.D.
