# Supplementary material for: Oncogenic Mutant p53 Sensitizes Non–Small Cell Lung Cancer Cells to Proteasome Inhibition via Oxidative Stress–Dependent Induction of Mitochondrial Apoptosis
Source: Cancer Res Commun. 2024 Oct 15;4(10):2685–98. doi: 10.1158/2767-9764.CRC-23-0637 (PMC11474859; doi:10.1158/2767-9764.CRC-23-0637)
Supplement: Figure S4 [file crc-23-0637_figure_s4_suppsf4.pdf]

Figure S4

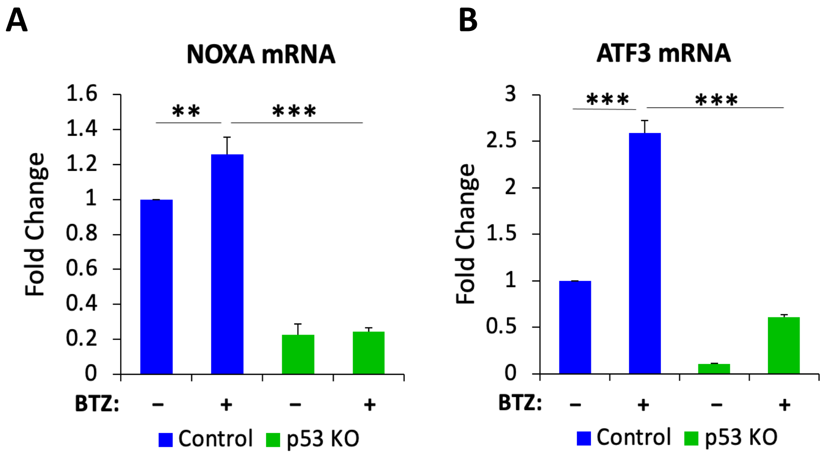

Fig. S4. BTZ-mediated ATF3/NOXA induction requires Onc-p53. A-B. H1975-Control or H1975-p53KO cells were treated with BTZ (5 nM) for 48 h and NOXA and ATF3 expression were analyzed by qRT-PCR. **p<0.01, ***p<0.005. Error bars indicate +/- 1.0 S.D.
