# Supplementary material for: Oncogenic Mutant p53 Sensitizes Non–Small Cell Lung Cancer Cells to Proteasome Inhibition via Oxidative Stress–Dependent Induction of Mitochondrial Apoptosis
Source: Cancer Res Commun. 2024 Oct 15;4(10):2685–98. doi: 10.1158/2767-9764.CRC-23-0637 (PMC11474859; doi:10.1158/2767-9764.CRC-23-0637)
Supplement: Figure S5 [file crc-23-0637_figure_s5_suppsf5.pdf]

Figure S5

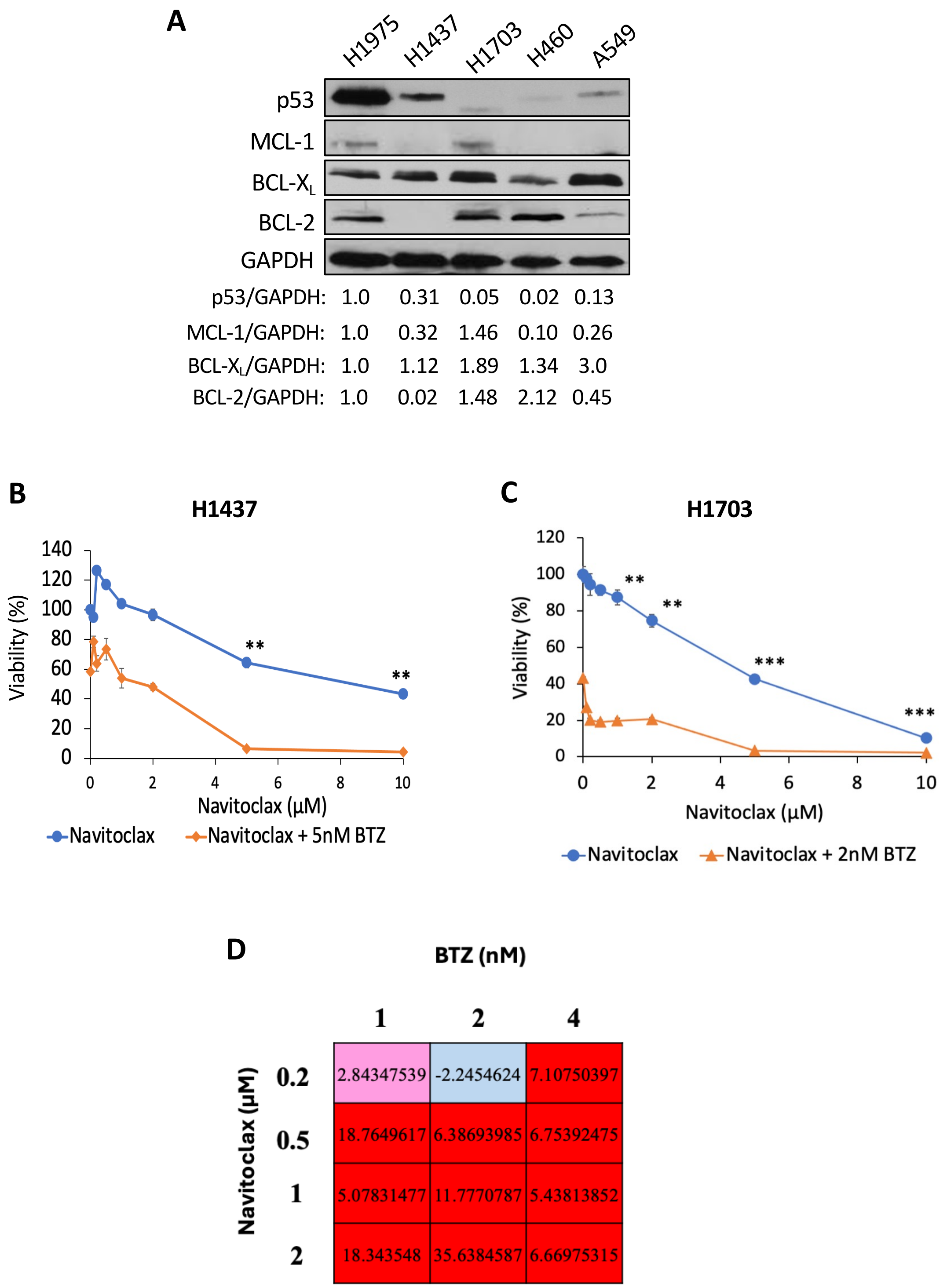

Fig. S5. Navitoclax enhances BTZ-induced cytotoxicity in Onc-p53-expressing NSCLC cells. A. Basal levels of anti-apoptotic MCL-1, BCL-X_L and BCL-2 proteins were determined by immunoblotting. **B.** H1437 and **C.** H1703 cells were treated with vehicle or the indicated concentrations of navitoclax with/without BTZ (5 nM) for 96 h. Cell viability was determined by WST-1 assay. ***p*<0.01; ***p<0.005. Error bars indicate +/- 1.0 S.D. **D.** A dose matrix of BTZ and navitoclax was performed on H1975 cells. The percent of the excess over the Bliss score at each dose combination is shown. Bliss scores greater than zero, close to zero, and less than zero represent synergy, additivity, and antagonism, respectively.
