# Supplementary material for: Oncogenic Mutant p53 Sensitizes Non–Small Cell Lung Cancer Cells to Proteasome Inhibition via Oxidative Stress–Dependent Induction of Mitochondrial Apoptosis
Source: Cancer Res Commun. 2024 Oct 15;4(10):2685–98. doi: 10.1158/2767-9764.CRC-23-0637 (PMC11474859; doi:10.1158/2767-9764.CRC-23-0637)
Supplement: Figure S6 [file crc-23-0637_figure_s6_suppsf6.pdf]

Figure S6

A

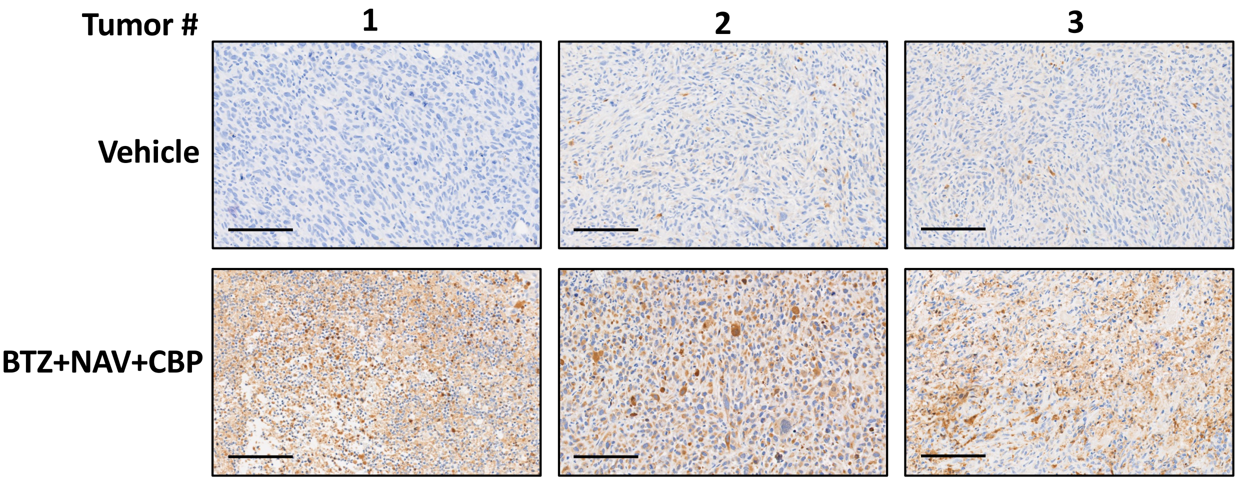

B

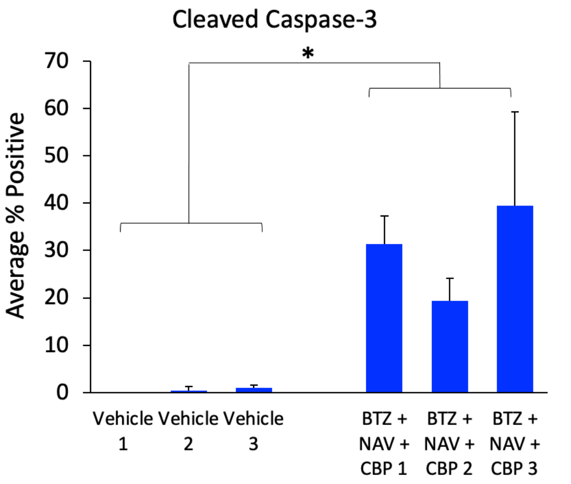

C

	Neutrophils (K/µL) (Mean ± SD)	Platelets (K/µL) (Mean ± SD)
**Vehicle**	1.81 ± 1.12	616.33 ± 400.89
**BTZ + NAV + CBP**	1.58 ± 0.68	996.50 ± 54.45
**Normal range**	0.6-3.16	651-1878

D

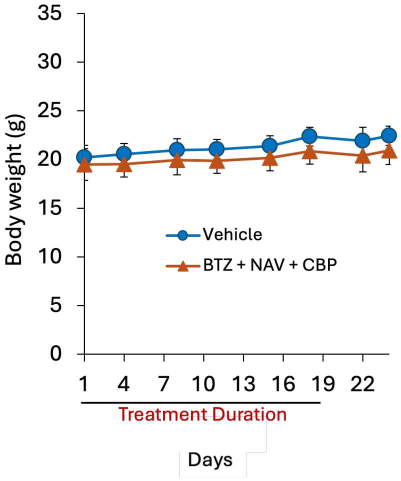

Fig. S6. Mechanism of action and toxicity of the BTZ + navitoclax + carboplatin regimen in H1975 xenografts. A. FFPE sections of H1975 xenografts obtained from mice treated with vehicle or BTZ + navitoclax (NAV) + carboplatin (CBP) combination (**Fig. 5D)** were stained by IHC with cleaved caspase-3 antibody. Photomicrographs are representative of 3 independent tumors from each treatment cohort. Scale bar = 50 µm, 20X magnification. **B.** The average percentage of cleaved caspase-3 positive cells in **A** were quantified from 5 random fields of each tumor section at 40X magnification. **C.** Circulating platelet and neutrophil counts obtained on day 22 from H1975-xenografted mice treated with vehicle vs. BTZ+NAV+CBP combination in **Fig. 5D**. Normal neutrophil and platelet count ranges are indicated. **D.** The average body weight of H1975-xenografted mice treated with vehicle vs. BTZ+NAV+CBP combination in **Fig. 5D**. *$p<0.05$. Error bars indicate +/- 1.0 S.D.
